# Supplementary material for: A near atomic structure of the active human apoptosome
Source: eLife. 2016 Oct 4;5:e17755. doi: 10.7554/eLife.17755 (PMC5050015; doi:10.7554/eLife.17755)
Supplement: Figure 1—source data 1. — Model statistics in the table were derived from an analysis with Molprobity (Davis et al., 2007). DOI: http://dx.doi.org/10.7554/eLife.17755.004 [file elife-17755-fig1-data1.doc]

**Figure 1--- source data 1. Data collection and refinement statistics**

| **Data Collection**  Microscope  Voltage (kV)  Detector  Electron Dose (e^-^/Å^2^)  2x binned pixel size (Å)  Defocus range (µm)  Movies | Titan Krios with energy filter  300  K2 Summit direct electron detector  40 (all frames)/37.8 (frames 2-18)  1.35  -1.5 to -2.4  4900 (18 frames) |
| --- | --- |
| **3D Reconstruction**  Software  Initial particles  Final particles  Symmetry  Resolution of unsharpened map (Å)  Resolution after particle polishing (Å)  B-factor for sharpening (Å^2^)  Resmap range (Å) | RELION and EMAN2  134970  92867  c7  4.5  4.1  -162  3.5-7 |
| **Model Statistics**  Ramachandran plot, favored (%)  Ramachandran plot, outlier (%)  Rotamer outlier  Molprobity score  Clash score  Bond length RMSD (Å)  Bond angle RMSD (^o^) | 93.18  0.3  0.51  2.13  15.19  0.0114  1.31 |
